# No Detectable Differences in microRNA Plasma Levels between Diabetic Hypertensive Patients with and without Incident Subclinical Atrial Fibrillation

**DOI:** 10.3390/jcm13092554

**Published:** 2024-04-26

**Authors:** Søren Feddersen, Tine J. Philippsen, Michael S. Hansen, Lene S. Christensen, Mads Nybo, Axel Brandes

**Affiliations:** 1 Department of Clinical Biochemistry, Odense University Hospital, J.B. Winsløws Vej 4, 5000 Odense, Denmark; soeren.feddersen@rsyd.dk (S.F.); mads.nybo@rsyd.dk (M.N.); 2 Department of Clinical Research, University of Southern Denmark, Campusvej 55, 5230 Odense, Denmark; 3 Department of Cardiology, Hospital of Southern Jutland, Kresten Philipsens Vej 15, 6200 Aabenraa, Denmark; tine.philippsen@rsyd.dk (T.J.P.); michael.skov.hansen@rsyd.dk (M.S.H.); lene.svendstrup@rsyd.dk (L.S.C.); 4 Department of Cardiology, Esbjerg Hospital—University Hospital of Southern Denmark, Finsensgade 35, 6700 Esbjerg, Denmark; 5 Department of Regional Health Research, University of Southern Denmark—Esbjerg, Finsensgade 35, 6700 Esbjerg, Denmark

**Keywords:** microRNA, subclinical atrial fibrillation, electrical remodelling, structural remodelling, insertable cardiac monitor

## Abstract

**Background**: Long-term rhythm monitoring (LTRM) can detect undiagnosed atrial fibrillation (AF) in patients at risk of AF and stroke. Circulating microRNAs (miRNAs), which have been shown to play a role in atrial electrical and structural remodelling, could help to select patients who would benefit most from LTRM. The aim of this study was to investigate whether patients with diabetes mellitus (DM) and hypertension and screen-detected subclinical AF (SCAF) using an insertable cardiac monitor (ICM) have significantly different plasma baseline levels of five selected miRNAs playing a role in the modulation of atrial electrical and structural remodelling (miR-21-5p, miR-29b-3p, miR-150-5p, miR-328-3p, and miR-432-5p) compared to those without SCAF. **Methods**: This study was performed at the outpatient clinic of a secondary academic teaching hospital between December 2013 and November 2015. Eligible patients were ≥65 years of age with DM and hypertension but without known heart diseases. All patients received an ICM. On the day of ICM implantation, blood samples for the measurement of plasma levels of the five miRNAs were drawn. In this post hoc analysis, we investigated their expression by reverse transcription-quantitative polymerase chain reaction. MiRNA plasma levels in patients with and without newly detected SCAF were compared. **Results**: We included 82 consecutive patients (median age of 71.3 years (IQR 67.4–75.1)), who were followed for a median of 588 days (IQR: 453–712 days). Seventeen patients (20.7%) had ICM-detected SCAF. Plasma levels of miR-328-3p, miR-29b-3p, miR-21-5p, miR-432-5p, and miR-150-5p were slightly but not significantly different in patients with incident SCAF compared with patients without. **Conclusions**: In patients with hypertension and DM, newly detected SCAF was not significantly associated with changes in expression levels of miR-21-5p, miR-29b-3p, miR-150-5p, miR-328-3p, and miR-432-5p.

## 1. Introduction

Atrial fibrillation (AF) is associated with increased mortality and cardiovascular morbidity, especially ischemic stroke, systemic thromboembolism, and heart failure [1,2]. If AF can already be detected at a subclinical state, early comprehensive treatment could prevent complications and progression [3,4,5]. However, subclinical AF is often paroxysmal and therefore difficult to detect by a single 12-lead ECG or short-term monitoring, which is why long-term cardiac rhythm monitoring is needed, which is invasive and relatively expensive. Circulating biomarkers could help in preselecting individuals, who would benefit most from long-term rhythm monitoring to detect AF.

B-type natriuretic peptide (BNP) and cardiac troponins have been widely investigated in AF [6,7,8,9,10,11,12,13]. Recently, circulating microRNAs (miRNAs) have been suggested as novel biomarkers in cardiovascular diseases including AF, as they are fairly stable and easily detected in plasma and serum [14,15]. MiRNAs are a class of small (~22 nucleotides) non-coding RNAs, which play important roles in regulating gene expression by hybridizing to target messenger RNAs and thereby regulating their translation or stability [16,17,18,19,20,21,22]. In human and animal studies, several miRNAs have been shown to play a role in the modulation of atrial electrical (miR-328-3p) and structural remodelling (miR-21-5p, miR-29b-3p, miR-150-5p, and miR-432-5p), thereby promoting and maintaining AF but also reducing vulnerability to AF [14,23,24,25,26,27,28,29,30]. A number of studies have demonstrated significantly different circulating miRNA levels in patients with clinically manifest AF compared to individuals without AF [26,27,29,30,31]. It has also been shown that ablation therapy to maintain sinus rhythm significantly changes miRNA plasma levels, so they become similar to those in individuals without AF [27,30]. On the other hand, data on the association between miRNA plasma levels and incident clinical AF are sparse and conflicting [31,32,33]. Moreover, only one study investigated the association between miRNA plasma levels and incident subclinical AF in a small cohort of patients with cryptogenic stroke undergoing continuous monitoring with an insertable cardiac monitor (ICM) [34]. The authors found that miR-1-3p plasma levels were significantly higher in patients with new subclinical AF.

The aim of this post hoc analysis was to investigate whether patients ≥65 years of age with diabetes mellitus and hypertension and screen-detected subclinical AF have significantly different plasma baseline levels of five miRNAs playing a role in the modulation of atrial electrical and structural remodelling (miR-21-5p, miR-29b-3p, miR-150-5p, miR-328-3p, and miR-432-5p) compared to those without screen-detected AF.

## 2. Materials and Methods

### 2.1. Study Population

This study was designed as a prospective single-centre observational study, carried out at a secondary academic teaching hospital. Patients ≥65 years of age with hypertension and diabetes mellitus, but without known or suspected AF, and attending the diabetic and cardiology outpatient clinic at the Hospital of Southern Jutland, Denmark, were included between December 2013 and November 2015. Details of the inclusion/exclusion process have previously been published [35]. Briefly, patients could be included if they were treated with at least two antihypertensive drugs and with any oral antidiabetics or insulin. All medications had to be stable for at least one month prior to inclusion. The following exclusion criteria applied: other risk factors for AF than hypertension and diabetes; known AF; ongoing oral anticoagulant (OAC) treatment; left ventricular ejection fraction <45%; valve disease requiring intervention; implanted pacemaker or implantable cardioverter defibrillator; known ischemic heart disease; stroke/transient ischemic attack and/or peripheral artery disease; thyrotoxicosis; and end-stage renal disease. This study was registered at ClinicalTrials.gov (NCT02041832) and complied with the Declaration of Helsinki. All patients gave written informed consent before enrolment. This study was approved by the Regional Scientific Ethical Committees of Southern Denmark (Project-ID S20130062) and the Danish Data Protection Agency (No. 13/12874).

### 2.2. Device Implantation and AF Definitions

An ICM (Reveal^®^ XT, Medtronic Inc., Minneapolis, MN, USA) was implanted subcutaneously in the left parasternal region in all the study participants. One patient received a Reveal LINQ^TM^ (Medtronic Inc., Minneapolis, MN, USA) after a minor pocket infection of the initially implanted Reveal^®^ XT. The detection method and AF definition have previously been described elsewhere [35]. In brief, the presence of AF was defined as at least one episode of absolutely irregular rhythm without P waves of ≥2 min duration, as the algorithm of the ICM classifies the rhythm for a time interval of at least 2 min in duration [36]. Incident subclinical AF was defined as new-onset ICM-detected AF without any symptoms.

### 2.3. RNA Extraction

Blood samples were drawn into K2-ethylenediaminetetraacetic acid (EDTA) tubes (BD Bioscience, Haryana, India), and within 30 min after sample collection, tubes were centrifuged at 2000× *g* for 20 min at 20 °C. Plasma was retrieved and stored at −80 °C. Total RNA, including small RNA, was subsequently extracted from 300 µL plasma samples using the mirVana PARIS kit (Life Technologies, Waltham, MA, USA) according to the manufacturer’s instructions for liquid samples. RNA was eluted with 100 µL of RNase free water into RNase-free tubes containing 1 µL RNase inhibitor. All samples were spiked with 5 fmol synthetic Arabidopsis thaliana miR-159a (ath-miR-159a).

### 2.4. Reverse Transcription-Quantitative Polymerase Chain Reaction (RT-qPCR)

Total RNA was converted to cDNA using the TaqMan MicroRNA Reverse Transcription (RT) Kit and miRNA-specific stem-loop RT primers from TaqMan MicroRNA Assays (Applied Biosystems, Thermo Fisher Scientific, Foster City, CA, USA). RT was performed in a 5 µL reaction containing 1.67 µL of the RNA extract, 0.05 µL of 100 mM dNTP (with dTTP), 0.33 µL of MultiScribe^TM^ reverse transcriptase (50 U/µL), 0.5 µL of 10× RT buffer, 0.063 µL of RNase inhibitor (20 U/µL), 1.387 µL of RNase-free water, and 1 µL of 5× miRNA-specific stem-loop RT primer (Applied Biosystems, Thermo Fisher Scientific, Foster City, CA, USA) as previously described [37,38]. RT reactions were performed in triplicate for each specific miRNA. To increase sensitivity, RT products were pre-amplified using TaqMan MicroRNA Assays (Applied Biosystems, Thermo Fisher Scientific, Foster City, CA, USA) diluted to 0.2× with TE buffer (pH 8.0) and TaqMan PreAmp Mastermix (Applied Biosystems, Thermo Fisher Scientific, Foster City, CA, USA). PreAmp reactions had a final volume of 7 µL and contained 1.75 µL RT product. Thermal cycling conditions were 95 °C for 10 min followed by 14 cycles of 95 °C for 15 s and 60 °C for 4 min.

For the quantitative PCR (qPCR) step, 1 µL of PreAmp product was mixed with TaqMan Universal PCR Master Mix II (2×) and TaqMan miRNA assay (20×) in a final volume of 10 µL. QPCR was performed either on a StepOne Plus or a ViiA7 real-time instrument (Applied Biosystems, Thermo Fisher Scientific, Foster City, CA, USA), and samples were run in duplicate. Thermal cycling conditions were 60 °C for 30 s and 95 °C for 10 min followed by 40 cycles of 95 °C for 15 s and 60 °C for 1 min. The StepOne Plus software (version 2.3) and the ViiA7 Real-Time qPCR analysis software (version 1.2) was used to obtain Cq-values, which were normalized to the synthetic miRNA ath-miR-159a. Exogenous normalized data were then exported to the qBase^PLUS^ software (version 3.0) (Biogazelle, NV, Ghent, Belgium) for inter-run calibration.

### 2.5. Normalization

Cq-values were normalized across samples using the median normalization procedure described by Mitchell et al. and the synthetic miRNA ath-miR-159a. The Cq-value for a given miRNA in a given sample was adjusted as follows: exogenous normalized Cq-value for the miRNA in the sample = Raw Cq-value for the miRNA in the sample—[(average ath-miR-159a Cq-value of the given sample)—(median ath-miR-159a Cq-value)] [37]. Exogenous normalized values were then exported to the qBase^PLUS^ software (version 3.0) (Biogazelle, NV, Belgium) for inter-run calibration. To measure miRNA levels, samples were spread across six different runs for each miRNA and data were corrected for inter-run variation using inter-run calibration. For each measured miRNA, two samples (inter-run calibrators) were measured in the six different runs in addition to the other samples that were spread across the runs. In the qBase^PLUS^ software (version 3.0), the results for the inter-run calibrators were used to quantify and correct for inter-run variation by determining a calibration factor for every miRNA-run combination [39].

### 2.6. Data Acquisition and Statistical Analyses

Data were extracted from the patients’ medical records, entered into a database (Excel 2010, Microsoft Corporation, Redmond, WA, USA) and analysed using STATA 14 (StataCorp LP, College Station, TX, USA). Categorical variables were presented as percentages and analysed using Fisher’s exact or χ^2^ test as appropriate. Continuous variables were presented as mean ± standard deviation (SD) if data followed a normal distribution, and Student’s t test was used to test for differences between groups. Non-Gaussian distributed continuous variables were reported as median with interquartile range (IQR), and Wilcoxon rank sum test was used. Statistical analyses of RT-qPCR data were performed using the GraphPad Prism (9.0.2 version) software (La Jolla, CA, USA). Student’s t test for unpaired data was used to test for differences between patients with and without screen-detected subclinical atrial fibrillation. For all analyses, a two-sided *p*-value < 0.05 was considered statistically significant.

## 3. Results

### 3.1. Characteristics of the Study Population

**Figure 1:**
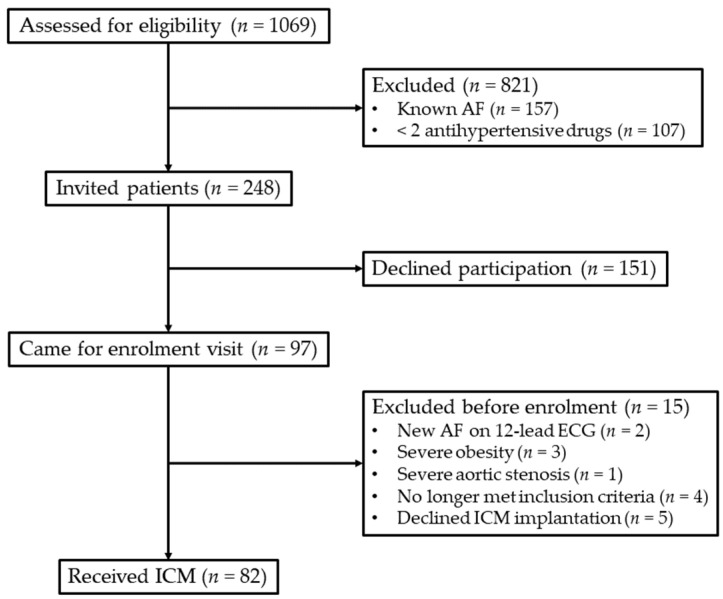
Screening and enrolment of the study cohort. AF, atrial fibrillation; ECG, electrocardiogram; ICM, insertable cardiac monitor.

A total of 248 patients were invited to participate, of whom 151 declined, mainly because they refused the implantation of the loop recorder. Another 15 patients were excluded for various reasons (Figure 1). Thus, the final population consisted of 82 patients with a median age of 71.3 years (IQR 67.4–75.1), who never had any symptoms suspicious of AF or other cardiovascular diseases. As shown in Table 1, there were no significant differences in age, sex distribution, body mass index, CHA_2_DS_2_-VASc score, medical treatment, laboratory, and echocardiographic parameters in patients with new subclinical AF compared with those without. They received on average four antihypertensive drugs without difference between groups. Diabetes mellitus and hypertension were relatively well managed in the long term in both groups as reflected by mean HbA1c values (Table 1) and according to the medical records; both groups also kept home blood pressure measurements.

During a median follow-up of 588 days (IQR: 453–712 days), 17 patients (20.7%) had screen-detected episodes of subclinical AF, all of which being completely asymptomatic. The median time from inclusion to the first-detected episode was 91 days (IQR 41–251 days). Three patients had subclinical AF episodes <6 min, two patients between 6 and 30 min, five patients between 30 min and 6 h, and seven patients between 6 h and ≥24 h. All patients with at least one episode of subclinical AF of ≥6 min duration initiated OAC treatment.

### 3.2. MicroRNA Findings

**Figure 2:**
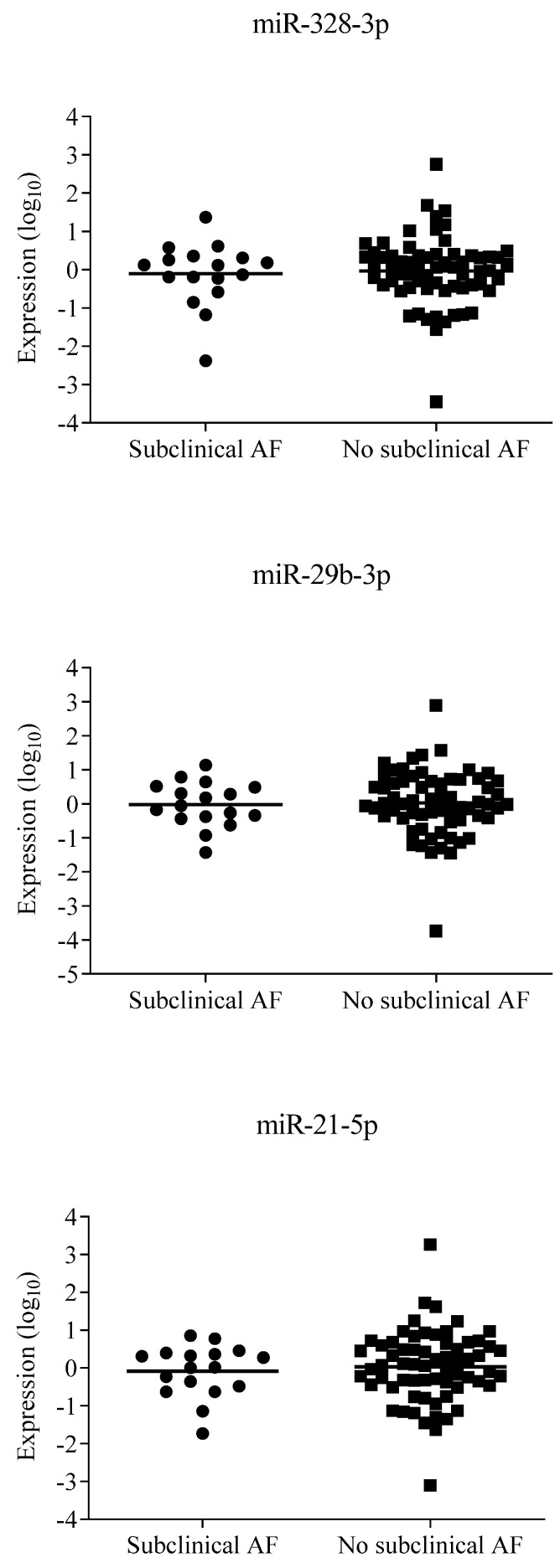

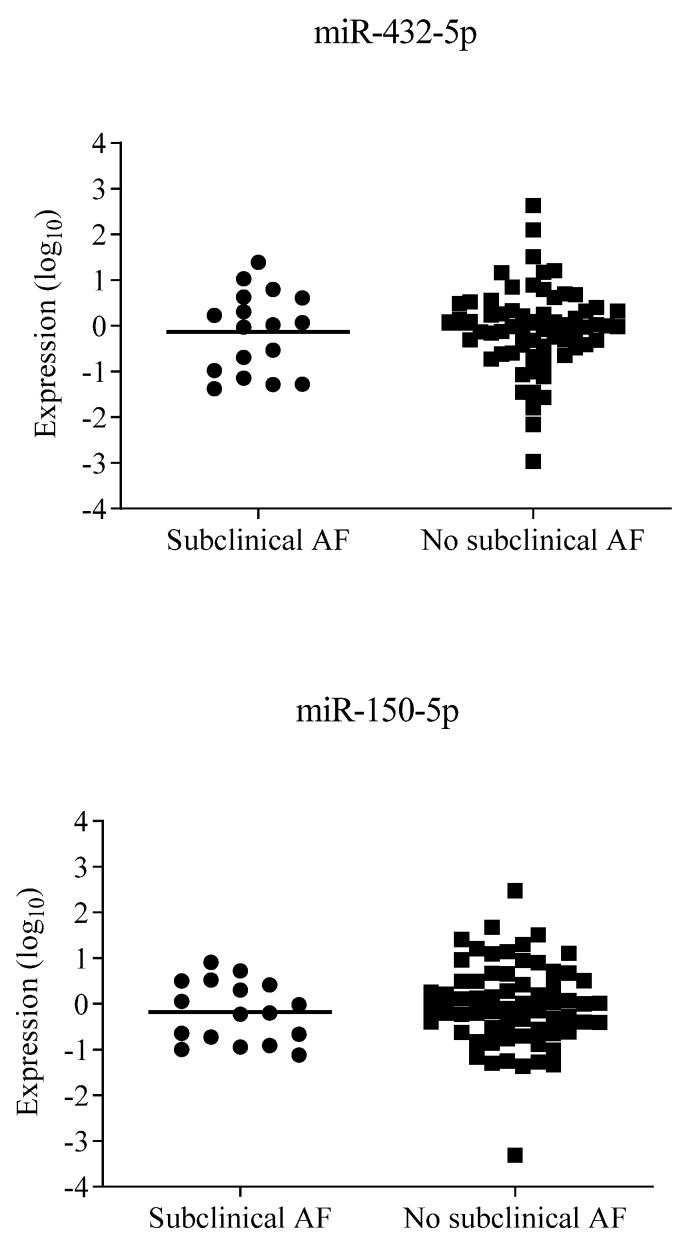
Plasma levels of microRNAs in patients with and without screen-detected subclinical atrial fibrillation. AF, atrial fibrillation.

Baseline plasma levels of the measured miRNAs miR-328-3p, miR-29b-3p, miR-21-5p, miR-432-5p, and miR-150-5p were slightly but not significantly different in patients with incident subclinical AF compared with patients without subclinical AF (Table 2; Figure 2).

## 4. Discussion

The main finding of this post hoc analysis was that patients with diabetes mellitus and hypertension, who have subclinical AF detected by long-term rhythm monitoring with an ICM, did not have significantly different plasma levels of miR-21-5p, miR-29b-3p, miR-150-5p, miR-328-3p, and miR-432-5p compared to patients without subclinical AF (Table 2; Figure 2).

To the best of our knowledge, the association between plasma levels of circulating miRNAs and incident subclinical AF in continuously monitored patients at high risk of AF and stroke has never been investigated.

Only one small study has looked at the association between miRNA plasma levels and subclinical AF so far: Benito et al. investigated a group of patients with cryptogenic stroke, who were continuously monitored by an ICM. They screened a large panel of 754 miRNAs and showed that only miR-1-3p, which regulates genes involved in arrhythmogenesis, was significantly associated with subclinical AF and modestly associated with AF burden [34].

Over the past decade, we have seen a growing body of evidence that non-coding RNAs including miRNAs play an important role in the development and pathophysiology of various diseases including arrhythmias but could also serve as novel biomarkers that may improve early recognition, treatment, and prognosis of patients with arrhythmias [40]. A number of human and animal studies have investigated the altered expression of miRNAs in heart tissue, which are regarded as closely related to the major pathophysiological mechanisms of AF including electrical, structural, and autonomic nervous remodelling as well as abnormalities in Ca^2+^-handling [40,41]. Moreover, circulating miRNAs may also have a more diagnostic and prognostic value [40,42].

Dawson et al. found that miR-29b plasma levels were significantly decreased in patients with clinical AF or chronic heart failure or both compared to controls, indicating that this miRNA would play a role in atrial structural remodelling [26]. In an analysis from the Framingham Offspring Study, McManus et al. measured the expression of 385 miRNAs in whole blood and found that only circulating levels of miR-328, a miRNA known to promote atrial electrical remodelling by diminishing L-type Ca^2+^ current [29], were associated with prevalent clinical AF. Individuals with known AF showed higher mean expression levels of miR-328 than individuals without. None of the measured miRNAs were, however, associated with incident AF. The authors explained the lacking association between miR-328 and incident AF by the limited number of new-onset AF cases in their study cohort [31]. In a recent work, the same group investigated the relationship between plasma miRNA levels, echocardiographic markers of atrial remodelling and incident clinical AF and found that six miRNAs associated with left atrial function index were also associated with clinical incident AF (miR-20a-5p, miR-26a-5p, miR-106b, miR-324-3p, mi-363-3p, and miR-484). These miRNAs are predicted to regulate genes involved in electrical and structural cardiac remodelling [32]. Geurts et al. measured plasma levels of a large panel of miRNAs in participants of the population-based Rotterdam Study and did not find any significant association with incident clinical AF [33].

Therapeutic interventions can also affect plasma levels of circulating miRNAs. In the miRhythm study, plasma levels of 86 miRNAs were investigated in 112 individuals with clinical AF and 99 without AF. Plasma levels of miR-21 and miR-150, which play a role in atrial electrical and structural remodelling, were significantly lower in individuals with AF compared to those without. In individuals with paroxysmal AF, plasma levels of these two miRNAs were lower compared to individuals with persistent AF. After ablation, plasma levels of both miRNAs significantly increased [27]. Similar results for miRNAs miR-409-3p and miR-432 were found in a study of Chinese AF patients undergoing catheter ablation for AF [30].

Although miRNAs may appear as attractive, new biomarkers for predicting AF—monitoring AF treatment effects, predicting major adverse events in AF patients, or characterizing the underlying mechanism of the development and progression of AF [26,27,30,31,32,33,34,43]—there are several factors currently limiting their use in daily clinical practice. A major problem is the lack of standardization of the miRNA analysis process, from obtaining the RNA over the purification of the material to the measurement including the normalization of the measured miRNA levels, which has impact on the results and makes them difficult to compare [26,27,30,31,32,33,34]. Although most studies investigating circulating miRNAs used plasma samples [26,27,30,32,33,34], some also used whole blood samples [31]. In studies using whole blood, the blood cells contribute significantly to circulating miRNA [44], making direct comparison with plasma studies difficult. Moreover, the anticoagulant used for preventing coagulation of the blood sample, the storage temperature, and the time for preparation of the plasma sample may influence the plasma miRNA levels [45]. The cohort size may also affect the results in a way that true effects can be missed, and the detected effects can be wrong in small sample size experiments, especially when performing high-throughput miRNA screens [46]. This could also explain why we did not find any significant difference between miRNA levels of patients with and without subclinical AF in the present study, even though we did not perform a high-throughput miRNA screen looking for a positive signal from the panel of analysed miRNAs, but investigated five selected miRNAs, which have been shown to play a role in atrial electrical and structural remodelling [14,23,24,25,26,27,28,29,30]. Another possible explanation for our neutral findings could be that atrial remodelling was not yet pronounced enough and, thus, had not led to detectable differences in miRNA expression at the time, before subclinical AF was detected by the ICM in our patient population without known heart disease. These findings are in line with findings from previous studies, which neither found associations between circulating miRNAs and incident clinical AF [31,33]. In contrast, a recent study in patients with cryptogenic stroke, who were screened for AF using an ICM, found that plasma levels of miR-1-3p were significantly associated with newly detected AF using a high-throughput miRNA screen [34]. In the future, efforts should be made to select distinct miRNAs and validate them in different populations to determine, whether they are suitable as biomarkers for incident AF.

### Limitations

This study has some limitations: First, it is a single-centre study of a relatively small cohort with hypertension and diabetes mellitus, but without known heart disease, why the results may not be generalizable. Because of the small cohort size, we could have missed differences in miRNA expression between patients with and without subclinical AF. Second, we have selected and only investigated five miRNAs associated with atrial electrical and structural remodelling instead of performing a high-throughput miRNA screen. On the other hand, our approach was hypothesis-driven and scientifically correct. Third, the measured expression levels of the miRNAs in our study were only normalized against a synthetic (exogenous) miRNA. The lack of normalization against both synthetic miRNA and endogenous miRNA may have influenced the results. Stable endogenous miRNA, which could have been suitable for normalization in our study population, does, however, not exist. Fourth, we did not perform serial measurements over time. All blood samples were drawn at baseline. MiRNA levels could have been different at the time of detection of subclinical AF. Fifth, due to the relatively small sample size, we were not able to perform an analysis of the miRNA levels according to different strata of HbA1c and blood pressure levels, as one might expect that the degree to which the disease is controlled could affect the expression of miRNAs.

## 5. Conclusions

In patients with hypertension and diabetes mellitus, we did not find a significant association between newly detected subclinical AF using an ICM and miR-21-5p, miR-29b-3p, miR-150-5p, miR-328-3p, and miR-432-5p, which have been shown to play a role in atrial electrical and structural remodelling in patients with clinically manifest AF. Future studies on miRNAs in patients with AF must carefully consider a number of preanalytical and analytical caveats and should also be aware of the study population in terms of size and generalizability.

## Figures and Tables

**Table 1:** Baseline characteristics of the study participants.

	Included Patients (*n* = 82)	Subclinical AF (*n* = 17)	No Subclinical AF (*n* = 65)	*p*-Value
**Clinical parameters and medications**				
Age (years)	71 ± 4	73 ± 5	71 ± 4	0.11
Men	52 (63)	12 (71)	40 (62)	0.49
BMI (kg/m^2^)	31 ± 5	30 ± 5	31 ± 5	0.55
CHA_2_DS_2_-VASc score	4 (3–4)	4 (3–4)	4 (3–4)	0.44
Cholesterol-lowering drugs	72 (88)	15 (88)	57 (88)	1.00
Insulin treatment	61 (74)	14 (82)	47 (72)	0.54
Oral antidiabetics	61 (74)	12 (71)	49 (75)	0.69
Beta-blockers	32 (39)	7 (41)	25 (38)	0.84
Diuretics	68 (83)	13 (76)	55 (85)	0.47
Calcium channel blockers	57 (70)	10 (59)	47 (72)	0.28
ACE-I/ARB	79 (96)	16 (94)	63 (97)	0.51
No. of antihypertensive drugs	4 (0.9)	4 (0.9)	4 (0.9)	0.81
**Laboratory and echocardiographic parameters**				
HbA1c (mmol/mol)	60 ± 11	62 ± 11	60 ± 12	0.37
Creatinine (µmol/L)	88 (71–117)	99 (91–109)	85 (71–117)	0.03
LVEF (%)	60 ± 7	61 ± 9	60 ± 7	0.58
LA diameter (mm)	36 ± 5	37 ± 5	35 ± 4	0.10
LA volume index (mL/m^2^)	30 ± 7	28 ± 7	30 ± 7	0.36
LA maximum volume biplane (mL)	60 ± 15	58 ± 16	60 ± 15	0.59
LA minimum volume biplane (mL)	22 ± 8	22 ± 7	23 ± 8	0.60
LA emptying fraction (%)	63 ± 8	63 ± 5	63 ± 8	0.76

Data are presented as mean ± SD, *n* (%), or median (interquartile range). Abbreviations: ACE-I, angiotensin-converting enzyme inhibitor; AF, atrial fibrillation; ARB, angiotensin receptor blocker; BMI, body mass index; HbA1c, glycated hemoglobin; LA, left atrial; LVEF, left ventricular ejection fraction.

**Table 2:** Reverse transcription-quantitative polymerase chain reaction analysis of selected miRNAs in plasma of the cohort.

MicroRNA	Assay Number	Reference	Subclinical AF (*n* = 17)	No Subclinical AF (*n* = 65)	*p*-Value
miR-328-3p	543	[31]	−0.106 ± 0.754	−0.031 ± 0.501	0.76
miR-29b-3p	413	[26]	−0.016 ± 0.398	0.031 ± 0.818	0.85
miR-21-5p	397	[27]	−0.085 ± 0.561	0.033 ± 0.488	0.63
miR-432-5p	1026	[30]	−0.131 ± 0.287	−0.056 ± 0.347	0.76
miR-150-5p	473	[27]	−0.176 ± 0.218	−0.005 ± 0.377	0.47

Data are presented as mean of log10 values ± SD. Normalization was performed by normalizing to the synthetic miRNA ath-miR-159a. Assay number refers to TaqMan microRNA assays (Applied Biosystems, Thermo Fisher Scientific, Foster City, CA, USA). Abbreviations: AF, atrial fibrillation.

## Data Availability

Data from this study will be accessible to interested parties upon reasonable request to soeren.feddersen@rsyd.dk. Data will be shared with researchers, whose proposed use has received approval. Access will be primarily granted for the replication of the results presented in our study. A signed data access agreement, compliant with regional legislation and data authority requirements, must be obtained prior to data release.
